# Genetic Parameter Estimates of Growth Curve and Feed Efficiency Traits in Japanese Quail

**DOI:** 10.3390/ani13111765

**Published:** 2023-05-26

**Authors:** Ebru Kaya Başar, Doğan Narinç

**Affiliations:** 1 Statistical Consulting Application and Research Center, Akdeniz University, Antalya 07100, Turkey; 2 Department of Animal Sciences, Akdeniz University, Antalya 07100, Turkey; dnarinc@akdeniz.edu.tr

**Keywords:** feed conversion efficiency, Gompertz growth model, heritability, genetic correlation, Bayesian inference

## Abstract

**Simple Summary:**

In poultry genetic improvement studies, the selection schedule evaluates many characteristics together. It is important for the geneticist to know the heritability of these characteristics and their genetic relationships. Heritability estimates for growth traits and feed efficiency traits, as well as genetic correlations between these traits, were determined in this study using Japanese quails, which are acknowledged as model animals for poultry species. Heritability estimates were found to be high for body weight traits, moderate to high for the Gompertz growth curve parameters, and moderate for feed efficiency traits. Negative and moderate genetic correlations were estimated between feed conversion efficiency traits and body weight traits, but no high genetic correlations were estimated between feed conversion efficiency traits and the Gompertz growth model’s asymptotic weight parameter. As a result, it was concluded that the β_0_ parameter of the growth curve might be beneficial in selection studies.

**Abstract:**

This study aimed to estimate heritabilities for weekly body weight traits, the Gompertz growth curve parameters, and feed efficiency characteristics, as well as genetic correlations among characteristics. A total of 700 Japanese quails with pedigree records were used in this study. Body weight and feed consumption were measured individually on a weekly basis. Using weekly body weight data, the growth model parameters were estimated for each bird using the Gompertz nonlinear regression model. Multi-trait variance-covariance matrices were obtained with Bayesian inference using the Gibbs sampler. While estimates of high heritability (0.59 to 0.61) were found for weekly body weight traits, estimates of moderate heritability (0.23 to 0.37) were determined for feed intake and feed conversion efficiency traits. The estimated heritabilities for the parameters of the Gompertz model and inflection point coordinates were moderate (0.37 to 0.47). While genetic correlations between feed intake and body weight characteristics were positive and moderate (0.28 to 0.49), the genetic correlations between feed conversion efficiency and body weight traits were positive and strong (0.52 to 0.83). It has been concluded that the moderate negative genetic relationship between feed conversion efficiency and body weight may constrain selection studies. Due to the weak genetic correlation between the asymptotic body weight parameter of the Gompertz model and the feed conversion efficiency, it is thought that the total genetic gain will be greater if the mature weight parameter is also used as a selection criterion in genetic improvement studies.

## 1. Introduction

Studies on the genetic improvement of poultry are typically conducted in two distinct phases. The first of these is to enhance the within-line selection of desired characteristics. In the first stage, additive genetic variation is utilized to increase related traits, and birds with the best breeding value for multiple characteristics are selected as the parents of the following generation. The second stage entails the crossing of improved lines. Multiple line crosses improve profitability when there is non-additive genetic variation for one or more traits contributing to the profit function, and consequently, commercial birds are the result of three- and four-way strain crosses [1,2]. Within-line selection can continue for many years, and the most challenging applications of poultry genetic improvement studies (genetic variance, covariance, and breeding value estimations for multiple traits) are conducted in the first stage. Various characteristics are employed as selection criteria for both dam lines and sire lines [3]. Today, genomic selection applications utilizing improved SNP chips specific to the species allow for more accurate estimations of breeding value, and the generation interval has been shortened [4,5]. Notwithstanding, for a considerable number of traits utilized in genetic improvement studies, it is necessary to know the genetic variations and the covariances between traits in the flock. It is also necessary to know the direction and magnitude of genetic relationships between a large number of characteristics that may be used as selection criteria. This is the only way for a geneticist to determine which traits should be improved in which flocks.

There have been studies conducted to improve the meat and egg production of Japanese quail [3,6,7,8]. Also accepted as a model animal for poultry breeding research is the Japanese quail, which has a very short generation interval (about three months), a genetic architecture quite comparable to chickens, and is simple to raise. Estimates of genetic parameters obtained from studies using Japanese quails provide important information for the establishment of index criteria in multi-trait selection research studies on other poultry species [9]. In previous studies, the heritabilities for growth, slaughter-carcass, meat quality, feed efficiency, egg production, egg quality, and reproductive characteristics in Japanese quails were estimated. Some of the genetic relationships between these characteristics have also been determined [10,11,12,13,14]. However, there are very few studies examining the genetic relationships between feed intake, feed conversion, and growth characteristics. It is difficult to determine the individual feed consumption of each bird, which is the primary cause of this situation. Increasing feed costs and difficulties in obtaining high-quality feed ingredients are two of the most important concerns influencing the economic feasibility of commercial poultry production. The competition between humans and livestock is intensifying, particularly for grains, which comprise more than half of poultry feed ingredients. Since feed expenses account for 65–70 percent of the cost of poultry production, and must be improved [15]. Energy derived from the feed is used for maintenance (basic body functions) and growth in birds. To evaluate feed efficiency, the feed conversion ratio and residual feed intake characteristics are utilized. The feed conversion ratio is the ability to convert feed to body weight in birds reared for fattening over a specific period of time [16]. Feed conversion efficiency (FCE), the inverse of feed conversion ratio, is used more often as it moves in the same direction as other yield traits (desired to increase the mean, whereas FCR needs to be decreased), especially in selection indexes. [17]. The residual feed intake (RFI) is another measure of feed efficiency, and it is defined as the difference between actual and predicted feed intake. Selection for low RFI and low FCE by phenotypic or genetic approaches is important to increase feed efficiency [15]. The aim of this study is to estimate the heritabilities of body weights, growth curve parameters, feed consumption, and feed conversion efficiency characteristics in a basic Japanese quail flock, as well as genetic correlations between these traits.

## 2. Materials and Methods

The experiment was conducted at the Department of Animal Science, Akdeniz University, Türkiye. The care and usage of birds complied with Turkish laws and regulations and was approved by the Ministry of Food, Agriculture, and Livestock (decision number 22875267-325.04.02-E.3751911) and Akdeniz University’s Animal Experiments Local Ethics Committee. Japanese quail (*Coturnix coturnix japonica*) were used as animal material in the research. A total of 700 chicks obtained from 40 male and 120 female quails in a breeder flock in the Akdeniz University livestock facility, which have not been genetically improved before, constituted the animal material of this study. Each egg collected from the breeder flock for 10 days was numbered according to their parents. The collected eggs were kept at 14 °C and 70% relative humidity in a chiller cabinet. Before the eggs were placed in the incubator, gradual preheating was applied. In a commercial incubator, the eggs were separated according to their parents and exposed to a temperature of 37.5 °C and a relative humidity of 55% for the first 14 days. During the last three days of incubation, the eggs were exposed to a temperature of 37.2 °C and a relative humidity of 70% in parental-designed hatching baskets. The newly hatched chicks were kept until they were dry, after which the wing numbers were attached. Thus, the pedigree records (there are 860 individuals with pedigree files) were generated, and weekly live weights and other measurements were taken by matching the pedigree records during the experiment. Quail chicks were housed in brooder cages (90 cm^2^/quail) for the first three weeks (each chick was raised in divided cages for a period of 21 days.); following sex determination in the third week, they were transferred to individual fattening cages (160 cm^2^/quail) where they remained until the age of 42 days. At 21 days of age, the presence (for females) or absence (for males) of speckled breast feathers was used to determine the gender of birds. A grower diet with 24% crude protein and 2900 kcal of metabolizable energy/kg/kg was employed for the first 21 days, followed by a fattening diet with 23% crude protein and 2800 kcal of metabolizable energy/kg. From hatching until the end of the trial, ad libitum feeding, water, and a 23 h per day lighting schedule were utilized.

To obtain the estimates of individual growth curve parameters, all quail were weighed weekly from hatching to 6 weeks of age using a digital scale (±0.01 g). Birds were fasted for 4 h before weighing. Due to technical limitations, the feed intake was measured in small groups (4–8 individuals consuming from the same feeder), and feed conversion efficiency was measured individually for a total of 700 chicks. Feed intake and FCE were determined weekly and recorded for 1, 2, 3, 4, 5, and 6 weeks. Using these data (feed intake and body weight gain), cumulative feed consumptions and cumulative feed conversion efficiencies for each bird were calculated for ages 5 and 6 weeks.

In many previous studies, it has been reported that the Gompertz function is the most compatible growth model for the quail species [10,18,19,20]. Therefore, the Gompertz nonlinear regression model (1) was used to estimate the growth curve of each quail.

(1) $$y_{t}=\beta_{0}e^{\left(-\beta_{1}e^{-\beta_{2}t}\right)}$$

where $y_{t}$ is the weight at age *t*, $\beta_{0}$ is the asymptotic (mature) weight parameter, $\beta_{1}$ is the scaling parameter (constant of integration), and $\beta_{2}$ is the instantaneous growth rate (per day) parameter [10]. The Gompertz model is characterized by an inflection point in a manner such that $\beta_{0}/e$ of the total growth occurs prior to it and the remainder occurring after. The coordinates of the point of inflection, age and weight at the inflection point (IPA and IPW, respectively) were obtained as follows:

(2) $$IPA=\beta_{0}/e$$

(3) $$IPW=ln\left(\beta_{1}\right)/\beta_{2}$$

The descriptive statistics and Kolmogorov–Smirnov normality tests of the traits were obtained using the UNIVARIATE procedure of SAS 9.4 statistical software.

The following linear mixed effects model for two traits (4) was used in the analysis:

(4) y=Xβ+Zu+e

where y is the vector of observations, **β** is a vector of fixed effects, and **u** is a vector of random genetic effects. **X** and **Z** are known design matrices relating phenotypic records to β and **u**, respectively. **e** is a vector of random errors. It is further specified that covariance matrix is equal to $R=R_{0}⊗I$, where **I** represent the identity matrix and denotes genetic covariance matrix, while the matrix G is equal to $G=G_{0}⊗A$, where **A** is the numerator relationship matrix.

Finally, it is assumed that y follows a multivariate normal distribution which can be denoted as $y\sim MVN\left(X\beta ,ZGZ^{’}+R\right).$

To perform Bayesian analysis, the likelihood function of **y** and prior distributions of β and a should be defined, as well as variance-covariance components $G_{0},R_{0}$.

$$p\left(y|\beta ,u,G,R\right)\propto {\left|ZGZ^{’}+R\right|}^{-1/2}\;⨉exp\left\{-\frac{1}{2}\left[\left(y-X\beta \right)’{\left(ZGZ^{’}+R\right)}^{-1}\left(y-X\beta \right)\right]\right\}$$

A non-informative prior was assumed for **β,** which is a vector of fixed effects. The prior distribution assumed for $G_{0}$ and $R_{0}$ was an Inverse Wishart distribution which is conjugate to multivariate normal distribution [21].

$$p\left(\beta \right)\propto constant$$

$$p\left(u|G_{0},A\right)\sim MVN\left(0,G_{0}⊗A\right)$$

$$p\left(G_{0}|\nu_{A},V_{A}\right)\sim IW\left(\nu_{A},V_{A}\right)$$

$$p\left(R_{0}|\nu_{E},V_{E}\right)\sim IW\left(\nu_{E},V_{E}\right)$$

$$p\left(u|G_{0},A\right)\propto$$

$$p\left(u|G_{0},A\right)\propto {\left(2\pi \right)}^{-k/2}{\left|\Sigma \right|}^{-1/2}exp\left\{-\frac{1}{2}\left(u\right)’\Sigma^{-1}\left(u\right)\right\}$$

$$p\left(G_{0}|\nu_{A},V_{A}\right)\propto {\left|G_{0}\right|}^{-\left(1/2\right)\left(\nu_{A}+k+1\right)}exp\left[-\frac{1}{2}tr\left(G_{0}^{-1}V_{A}^{-1}\right)\right]$$

$$p\left(R_{0}|\nu_{E},V_{E}\right)\propto {\left|R_{0}\right|}^{-\left(1/2\right)\left(\nu_{E}+k+1\right)}exp\left[-\frac{1}{2}tr\left(R_{0}^{-1}V_{E}^{-1}\right)\right]$$

where $\Sigma =G_{0}⊗A$ and *k* = 2 for a multivariate model. We can write the joint posterior density of all parameters as

$$p\left(\beta ,a,G_{0},R_{0}|y\right)\propto p\left(y|\beta ,a,R_{0}\right)p\left(a|G_{0}\right)p\left(G_{0}\right)p\left(R_{0}\right)$$

Bayesian analyses were carried out using the MCMCglmm package of R [22]. A single sampling chain of 110,000 iterations was considered with 10,000 cycles of burn-in and a thinning interval of 50 cycles to obtain 2000 samples of the parameters of interest in total. Heritabilities ($h_{i}^{2}$) and genetic correlations ($r_{g\left(ii^{’}\right)}$), were calculated from the variance and covariance estimates as follows:

(5) $$h_{i}^{2}=\frac{\sigma_{ia}^{2}}{\sigma_{ia}^{2}+\sigma_{ie}^{2}}$$

(6) $$r_{g\left(ii^{’}\right)}=\frac{\sigma_{ii^{’}a}}{\sigma_{ia}^{2}+\sigma_{i^{’}a}^{2}}$$

where *i* and $i^{’}$ represents the trait(s) of interest, and $\sigma_{ia}^{2}$ and $\sigma_{ie}^{2}$ are the diagonal elements of $G_{0}$ and $R_{0}$ matrices, respectively. Additionally, $\sigma_{ii^{’}a}$ stands for the additive genetic covariance between the traits *i* and $i^{’}$.

## 3. Results

### 3.1. Basic Statistics

The descriptive statistics of BW5, BW6, FI5, FI6, FCE5, FCE6, β_0_, β_1_, β_2_, IPT, and IPW are presented in Table 1. At 5 and 6 weeks of age, females had greater mean body weight and cumulative feed intake values than males (all *p* < 0.05). At 5 and 6 weeks of age, the mean body weights of male quails were 181.88 g and 201.57 g, while the mean body weights of female quails were 191.78 g and 211.16 g, respectively. At 5 weeks of age, the average cumulative feed intake value for male quails was 431.45 g, whereas the average for female quails was 470.98 g. Similarly, the average cumulative feed intakes of male and female quails at 6 weeks of age were 595.88 g and 652.74 g, respectively. There were no statistical differences between the average cumulative feed conversion efficiencies of female and male quails at 5 and 6 weeks of age (both *p* > 0.05). At 6 weeks old, male and female quails had mean cumulative feed conversion ratios (1/FCE) of 2.46 and 2.43, respectively; at 6 weeks old, these averages had increased to 3.01 and 3.08, respectively.

**Figure 1:**
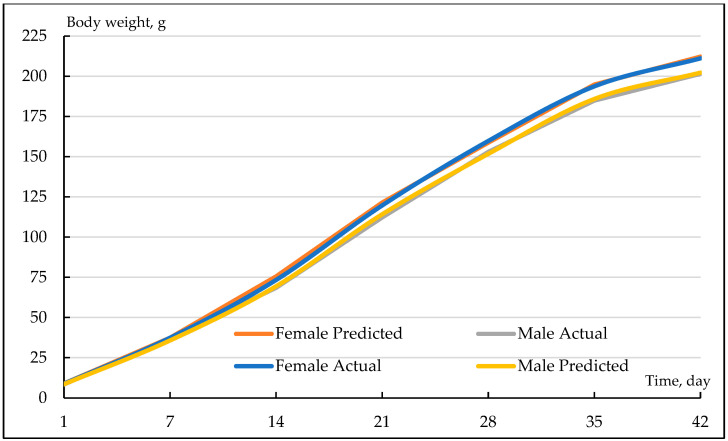
Female and male quail growth curves derived using the Gompertz model.

Figure 1 shows a graphical representation of the growth curves obtained by applying the Gompertz model to growth samples from female and male quails. As a result of nonlinear regression analyses utilizing the weekly individual body weights of all quails with the Gompertz growth model, coefficients of determination (R^2^) in the range of 0.9978 to 0.9999 were found. In terms of the distant asymptotic weight parameter (β_0_) of the Gompertz function, a statistically significant difference was found between male and female quails (*p* < 0.05). While the average β_0_ parameter of males was found to be 235.94 g, the mean value of the β_0_ parameter of females was estimated to be 260.01 g. Males and females did not differ statistically in terms of the scaling parameter and the instantaneous growth rate parameter of the Gompertz model (both *p* > 0.05). The mean values of β_1_ and β_2_ for male quails were estimated to be 3.29 and 0.068, while those for females were 3.32 and 0.071. Statistical differences were identified between genders in terms of inflection point coordinates of the Gompertz model (*p* > 0.05). The estimated age of inflection is younger for females (16.90 days) than for males (18.51 days). Likewise, the average inflection point weight of female quails (95.65) was found to be greater than that of males (86.81 g).

### 3.2. Heritability Estimates

**Figure 2:**
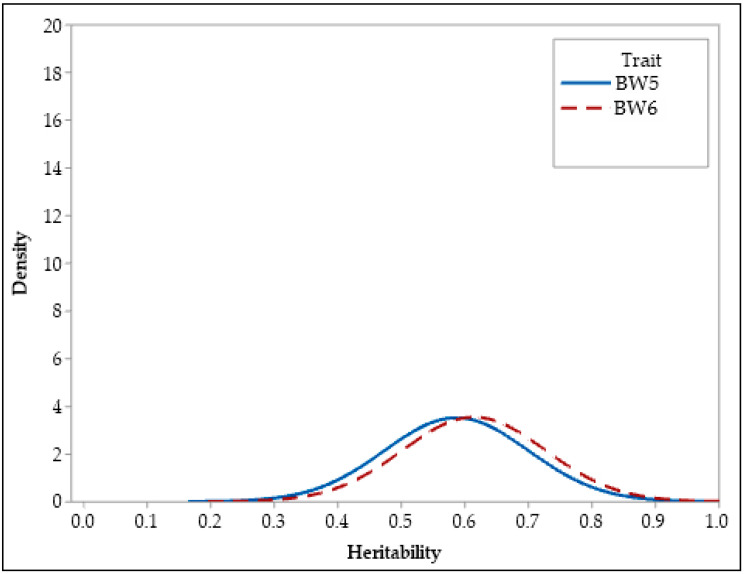
Posterior distributions of heritability of body weight traits.

**Figure 3:**
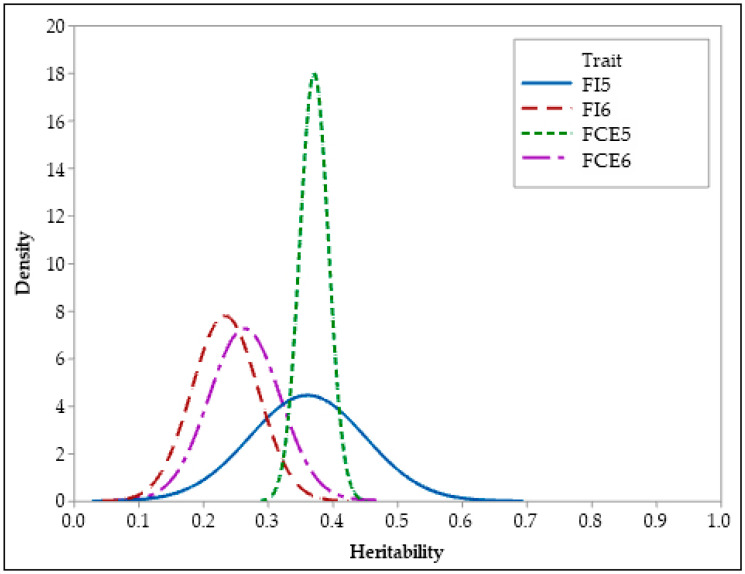
Posterior distributions of heritability of feed efficiency traits.

**Figure 4:**
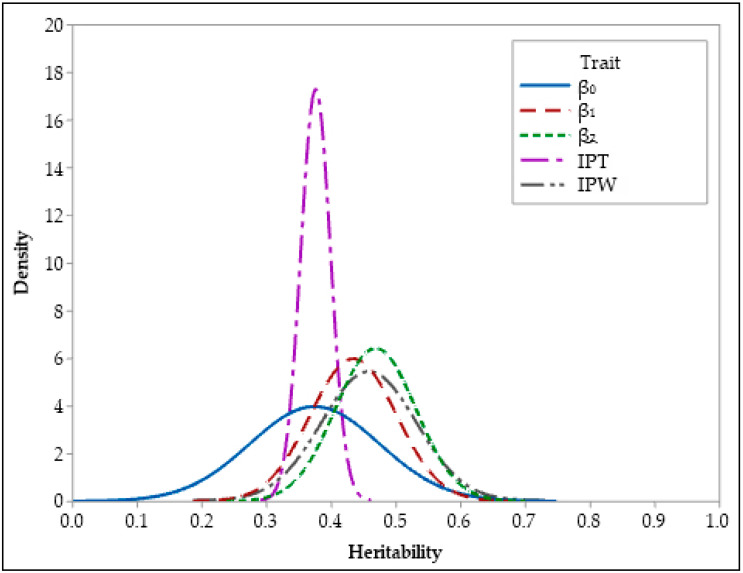
Posterior distributions of heritability of growth curve traits.

Summary statistics for the posterior distributions of heritability estimates of growth and feed efficiency traits are presented in Table 2, along with their posterior densities in Figure 2, Figure 3 and Figure 4. Both BCI and HPDI of the heritabilities in Table 2 are nearly identical and represent the same interval for the parameters of interest. Heritability estimates were found to be high (0.43–0.61) for BW5, BW6, β_1_, β_2_, and IPW characteristics. Heritability estimates for FI5, FCE5, β_0_, and IPT traits were moderate to high (0.36–0.37). In addition, estimations of the heritability of FI6 and FCE6 characteristics (respectively, 0.23 and 0.26) ranged from low to moderate.

### 3.3. Genetic and Phenotypic Relationships

Table 3 displays the genetic and phenotypic correlations between the traits studied in this research. Between BW5 and BW6, positive and strong genetic and phenotypic correlations (0.92 and 0.90, respectively) were estimated. The genetic and phenotypic correlations between feed intake and body weight characteristics were positive and moderate (0.28–0.49). In addition, genetic and phenotypic correlations between feed conversion efficiency and body weight traits were negative and strong. In this study, negative and moderate to high genetic and phenotypic relationships (ranging from −0.32 to −0.58) between feed intake and feed conversion efficiency characteristics were determined. Due to the nature of the Gompertz equation (IPW = β_0_/e and IPT = ln(β_1_)/β_2_), there are fixed relationships between parameter β_0_ and IPW and between IPT and parameters β_1_ and β_2_. Although the β_0_ parameter of the Gompertz model exhibited strong genetic and phenotypic relationships with BW5 and BW6 (0.52–0.82), no significant correlations were observed with feed efficiency characteristics. The phenotypic and genetic relationships between parameters β_1_ and β_2_ of the Gompertz model with body weight and feed efficiency characteristics were weak and statistically insignificant (ranging from −0.19 to 0.20).

## 4. Discussion

The average 5-week-old weight value for Japanese quails in this study is consistent with the findings of some researchers [14,23,24,25,26], who found the 5-week-old weight of the quails to be between 179.07 g and 190.00 g, which has not been conducted in any genetic improvement study before. Likewise, the mean value for 6-week-old weight found in this study is consistent with the values (200.13–232.4) reported by Lotfi et al. [27], Karaman et al. [28], and Shafik et al. [29]. Due to the difficulty in determining the individual feed intake of Japanese quail chicks, numerous studies [8,24,30] have only included feed efficiency traits between certain weeks. There are no reports in the literature about cumulative feed intake and cumulative feed conversion efficiency from hatch to weeks five or six. Additionally, Varkoohi et al. [24] reported that Japanese quails consumed 378 g of feed between 7 and 28 days, with a feed conversion efficiency of 0.40. Similarly, Foomani et al. [24] reported that Japanese quails consumed 345.12 g of feed between 0 and 28 days, with a feed conversion efficiency of 0.38.

Estimates of the Gompertz growth curve were produced for all birds with high determination coefficients (0.9978–0.9999). This indicates that the Gompertz function effectively explained the growth curves of the observed data, in accordance with Akbaş and Oguz [19] and Narinc et al. [31]. The mean value of the β_0_ parameter (249.91 g) was in agreement with the mean values reported by Beiki et al. [32] and Narinç et al. [13]. Similarly, the mean values for the β_0_ parameter of the Gompertz function in Japanese quails were estimated in the range of 242–276 g by Karabağ et al. and Hyankova et al. [14,33]. The integration coefficient parameter (β_1_) of the Gompertz model for the growth of Japanese quail was estimated by Akbaş and Yaylak [10] and Narinc et al. [31] to be 3.39 and 3.31, respectively, which is consistent with the mean value (3.31) in our study. The estimated mean values for the β2 parameter and inflection point coordinates of the growth model are consistent with the results of a number of studies [9,19,27,28] employing the Gompertz growth model in Japanese quails.

It has also been reported in previous studies that females have higher averages than males in terms of weekly body weights [34,35], feed intakes [11,36], β_0_ parameter of the growth model [14] and inflection point coordinates [20].

The Bayesian approach replaces frequentist credible intervals with Bayesian credible intervals (BCI). The interpretation of a 95% BCI is that there is a 0.05 statistical probability that the parameter falls inside the specified interval. Additionally, if the posterior distribution is asymptotic, the highest probability density interval (HPD), the shortest possible interval containing 95% of the posterior mass, is selected by Waldmann and Ericsson [37]. The overlap between the BC and HPD intervals of heritabilities in this study indicates that the estimates are reliable.

The heritability estimates for body weight at 5 and 6 weeks were 0.59 and 0.61, respectively (Table 2). Numerous researchers have observed high heritability estimates (0.46–0.69) for both characteristics [10,18,38,39], which are consistent with the findings of this study. As mentioned previously, there are few studies on the genetics of feed efficiency in Japanese quail, and these studies only assessed feed intake and feed conversion characteristics at short time intervals. In a study conducted by Akşit et al. [11], the heritability estimates of feed intake and feed conversion efficiency characteristics of quails (between 3- and 5-week-old) were found to be 0.53 and 0.39, respectively. Caetano et al. [40] reported that the heritability estimates of weekly feed conversion traits in two distinct quail flocks at 4 and 5 weeks of age were moderate to high (0.20–0.57). According to the study conducted by Foomani et al. [24], the heritability estimates of feed intake and feed conversion efficiency traits measured in quails between 0 and 28 days were 0.31 and 0.26, respectively. In another study [8], the heritability estimates of feed intake and feed conversion efficiency characteristics measured from 7 to 28 days of age in Japanese quails were found to be moderate. Individually recorded feed intake and feed conversion efficiency in chickens were found to have heritabilities of 0.48 and 0.49 in week 5 and 0.46 and 0.41 in week 6, respectively [34]. Moderate to high heritability values estimated by many researchers for feed intake and feed conversion efficiency in quail and chickens were found to be compatible with the results of this study.

In agreement with this study, [13,18,31] found moderate to high heritability estimates (0.41 and 0.38, respectively) for the β_0_ parameter. In addition, there are researchers who find the estimates of heritability for the β_0_ parameter to be both lower (0.17) [38] and higher (0.54) [41]. The high heritability estimates for the β_1_ and β_1_ parameters of the Gompertz model in this study are consistent with the findings of Saghi and Saghi [42] and Narinc et al. [13]. Various researchers have reported varying heritability estimates for the inflection point age of the Gompertz model in Japanese quails. Some researchers [14,18,31] have claimed that the heritability estimates of the IPT trait are low (0.08–0.21). The estimated heritability for the IPT trait in this study is comparable to moderate to high estimates (0.23–0.41) reported by Akbaş and Yaylak [10]. Because of the fact that the inflection point weight of the Gompertz growth model is obtained by dividing the β_0_ parameter by number e, all the variance components are equal to the β_0_ parameter. Due to the nature of the Bayesian estimator (Gibbs sampler) utilized in this study, only small iteration-related differences between the variance component estimates of β_0_ and IPW occurred.

It has been reported by many researchers that there are positive and high genetic and phenotypic correlations between the live weight characteristics of close weeks in Japanese quails [38,43,44,45]. Some studies have focused on the genetic relationships between body weight gain and feed conversion rate [34,46]. Moreover, some researchers have utilized the adjusted body weight characteristic instead of body weight gain to overcome this issue [11,47]. Since body weight gain is utilized to calculate feed conversion, it is expected that there will already be a considerable relationship between the two traits. Instead, it is more precise to evaluate the relationships between body weight traits and feed efficiency characteristics, which are currently selection criteria in poultry genetic improvement studies. There are very few studies evaluating the genetic relationships between feed efficiency traits and other yield-related characteristics in the scientific literature, including all poultry species. According to Akşit et al. [11], when quails consumed more feed, their residual feed consumption increased, and their feed efficiency declined. The researchers stated that, as a result, the birds utilized their feed sources less efficiently. Negative strong genetic correlations (ranging from −0.32 to −0.58) between FI5, FI6, and FCE5, FCE6 in this study support the findings of [11]. Koerhuis and Hill [48] estimated a weak genetic correlation of −0.20 between the feed conversion rate and body weight of broilers. The inverse of the feed conversion ratio is feed conversion efficiency. In agreement with Koerhuis and Hill [48], the phenotypic and genetic correlations between FCE and BW in our study were positive but rather strong. Positive genetic relationships estimated between FCE traits and BW traits in this study were also observed between FCE traits and the β_0_ parameter of the Gompertz growth model. Nevertheless, the genetic correlations (0.28 and 0.34) between β_0_ and FCE characteristics were relatively weaker than those between FCE and BW (ranging from 0.52 to 0.82).

## 5. Conclusions

In this study, it was determined that the heritability estimates for feed conversion traits were moderate, but considering the genetic relationships with other yield traits, using the FCE trait as a selection criterion would be more beneficial than the FI trait. In addition, it has been determined that the strong and positive genetic relationships between FCE traits and BW traits can have a very beneficial effect in multi-trait selection studies. Finally, due to the moderate genetic correlation between the β_0_ parameter of the Gompertz growth model and the FCE traits, it has been determined that the β_0_ parameter can be used as an alternative in case the additive genetic variance for live weight decreases in the following generations of selection.

## Figures and Tables

**Table 1:** Descriptive statistics for growth and feed efficiency traits ^1^.

Trait	Mean	Standard Deviation	Coefficient of Variance (%)	Minimum	Maximum	Sex Effect ^2^
BW5	185.84	19.23	10.35	134.89	242.90	0.001
BW6	204.54	20.48	10.01	150.22	277.83	0.001
FI5	449.77	51.89	11.54	291.59	614.51	0.001
FI6	621.48	77.74	12.51	354.52	822.28	0.001
FCE5	0.409	0.0576	14.09	0.254	0.608	0.078
FCE6	0.328	0.0507	15.44	0.211	0.466	0.126
β_0_	249.91	36.64	14.66	171.08	338.44	0.003
β_1_	3.31	0.23	6.84	2.77	3.99	0.125
β_2_	0.069	0.010	13.89	0.040	0.096	0.245
IPT	17.60	2.58	14.68	12.01	31.61	0.001
IPW	91.94	13.48	14.66	62.94	124.50	0.003

^1^ BW5–6 = Body weights at 5 and 6 weeks of age; FI5–6 = Cumulative feed intakes at 5 and 6 weeks of age; FCE5–6 = Cumulative feed conversion efficiencies at 5 and 6 weeks of age; β_0_ = Asymptotic body weight parameter; β_1_ = Shape parameter; β_2_ = Instantaneous growth rate parameter; IPT = Time at the inflection point of growth curve; and IPW = Body weight at the inflection point of the growth curve. ^2^ The *p* values of the Independent Sample *t*-test were evaluated at a significant level of 0.05.

**Table 2:** Posterior expectations, SD, credible intervals, and highest posterior density intervals of the heritability estimates.

Trait ^1^	Mean	Median	SD ^2^	MCSE ^3^	BCI ^4^ 2.5	BCI 97.5	HPDI ^5^ 2.5	HPDI 97.5
BW5	0.59	0.57	0.11	0.001	0.45	0.73	0.46	0.73
BW6	0.61	0.60	0.11	0.001	0.48	0.75	0.47	0.75
FI5	0.36	0.35	0.09	0.001	0.26	0.48	0.27	0.49
FI6	0.23	0.23	0.05	0.001	0.11	0.32	0.12	0.35
FCE5	0.37	0.37	0.02	0.001	0.25	0.49	0.29	0.51
FCE6	0.26	0.26	0.05	0.002	0.14	0.38	0.18	0.37
β_0_	0.37	0.36	0.10	0.001	0.25	0.49	0.28	0.51
β_1_	0.43	0.43	0.07	0.001	0.31	0.55	0.33	0.55
β_2_	0.47	0.47	0.06	0.001	0.36	0.58	0.36	0.61
IPT	0.37	0.37	0.02	0.001	0.27	0.50	0.26	0.49
IPW	0.38	0.37	0.10	0.001	0.27	0.48	0.29	0.50

^1^ BW5–6 = Body weights at 5 and 6 weeks of age; FI5–6 = Cumulative feed intakes at 5 and 6 weeks of age; FCE5–6 = Cumulative feed conversion efficiencies at 5 and 6 weeks of age; β_0_ = Asymptotic body weight parameter; β_1_ = Shape parameter; β_2_ = Instantaneous growth rate parameter; IPT = Time at the inflection point of growth curve; and IPW = Body weight at the inflection point of the growth curve. ^2^ The *p* values of the Independent Sample *t*-test were evaluated at a significance level of 0.05. SD = Standard deviation. ^3^ MCSE = Monte Carlo Standard errors, ^4^ BCI = Bayesian credible interval (2.5%, lower bound; 97.5%, upper bound). ^5^ HPDI = Highest posterior density interval (2.5%, lower bound; 97.5%, upper bound).

**Table 3:** The genetic correlation estimates (below diagonal) and phenotypic correlations (above diagonal) for growth and feed efficiency traits ^1^.

	BW5	BW6	FI5	FI6	FCE5	FCE6	β_0_	β_1_	β_2_	IPT	IPW
BW5		0.90 *	0.40 *	0.33 *	0.67 *	0.49 *	0.52 *	−0.11	−0.13	0.18	0.53 *
BW6	0.92 (0.01) ^2^		0.28 *	0.43 *	0.59 *	0.75 *	0.76 *	−0.09	−0.18	0.12	0.75 *
FI5	0.49 (0.01)	0.34 (0.01)		0.68 *	−0.49 *	−0.29 *	0.08	−0.02	−0.02	0.08	0.09
FI6	0.35 (0.11)	0.48 (0.01)	0.75 (0.07)		−0.29 *	−0.44 *	0.09	−0.01	−0.01	0.10	0.08
FCE5	0.71 (0.07)	0.61 (0.08)	−0.53 (0.08)	−0.32 (0.05)		0.78 *	0.29 *	0.19	−0.09	0.15	0.29
FCE6	0.52 (0.05)	0.83 (0.03)	−0.45 (0.03)	−0.58 (0.02)	0.83 (0.06)		0.27 *	0.06	−0.23	0.11	0.28
β_0_	0.58 (0.01)	0.82 (0.01)	0.16 (0.05)	0.12 (0.06)	0.34 (0.08)	0.28 (0.02)		−0.34 *	−0.64 *	0.66 *	0.99 *
β_1_	−0.17 (0.02)	−0.17 (0.03)	0.06 (0.05)	0.21 (0.04)	0.20 (0.05)	0.12 (0.02)	−0.37 (0.01)		0.51 *	0.71 *	−0.34 *
β_2_	−0.16 (0.02)	−0.19 (0.04)	0.13 (0.01)	0.11 (0.07)	0.12 (0.06)	0.14 (0.04)	−0.70 (0.02)	0.66 (0.01)		−0.89 *	−0.64 *
IPT	0.22 (0.04)	0.20 (0.03)	0.12 (0.04)	0.14 (0.04)	0.17 (0.01)	0.12 (0.06)	0.69 (0.07)	0.79 (0.01)	−0.95 (0.01)		0.66 *
IPW	0.58 (0.01)	0.82 (0.01)	0.15 (0.08)	0.22 (0.06)	0.34 (0.03)	0.29 (0.03)	0.99 (0.01)	−0.36 (0.06)	−0.71 (0.01)	0.70 (0.01)	

^1^ BW5–6 = Body weights at 5 and 6 weeks of age; FI5–6 = Cumulative feed intakes at 5 and 6 weeks of age; FCE5–6 = Cumulative feed conversion efficiencies at 5 and 6 weeks of age; β_0_ = Asymptotic body weight parameter; β_1_ = Shape parameter; β_2_ = Instantaneous growth rate parameter; IPT = Time at the inflection point of growth curve; and IPW = Body weight at the inflection point of the growth curve. ^2^ The standard error of the genetic correlation estimate is in parentheses. * The phenotypic correlation was statistically significant, *p* < 0.05.

## Data Availability

All data generated or analyzed during this study are included in this published article. The datasets used and/or analyzed in this study are available from the corresponding author upon reasonable request.
